# Human umbilical cord-derived mesenchymal stem cell therapy ameliorates lupus through increasing CD4+ T cell senescence via MiR-199a-5p/Sirt1/p53 axis

**DOI:** 10.7150/thno.48080

**Published:** 2021-01-01

**Authors:** Tao Cheng, Shuai Ding, Shanshan Liu, Yan Li, Lingyun Sun

**Affiliations:** 1 Department of Rheumatology and Immunology, The Affiliated Drum Tower Hospital, Medical School of Nanjing University, Nanjing, 210008 China; 2 The State Key Laboratory of Pharmaceutical Biotechnology, Chemistry and Biomedicine Innovation Center, Model Animal Research Center of Nanjing University, Nanjing, 210061 China

**Keywords:** Mesenchymal stem cells, Lupus, Senescence, Sirt1/p53, miR-199a-5p

## Abstract

**Rationale**: Although human umbilical cord-derived mesenchymal stem cells (hUC-MSCs) transplantation has been proved to be an effective therapeutic approach to treat systemic lupus erythematosus (SLE), the detailed underlying mechanisms are not fully understood. Transferring miRNAs is one mean by which MSCs communicate with surrounding cells. Sirt1 is a NAD-dependent deacetylase that protects against cell senescence by deacetylating p53. Here we aimed to explore whether hUC-MSCs affected senescence of splenic CD4+ T cells through regulating Sirt1/p53 via miRNA in the MRL/*lpr* lupus mouse model.

**Methods**: The effects of hUC-MSCs on lupus syndrome and senescence pathways in MRL/*lpr* mice *in vivo* and *in vitro* were determined. The functional roles of miR-199a-5p in splenic CD4+ T cell senescence were studied by miRNA mimic or inhibitor *in vitro.* MRL*/lpr* mice were injected with miR-199a-5p agomir to evaluate the effects of miR-199a-5p on splenic CD4+ T cell senescence and disease *in vivo.*

**Results**: We showed that hUC-MSCs transplantation ameliorated lupus symptoms and increased senescence of splenic CD4+ T cells through Sirt1/p53 signaling via miR-199a-5p in MRL/*lpr* mice. Moreover, systemic delivery of miR-199a-5p in MRL/*lpr* mice increased splenic CD4+ T-cell senescence, mimicking the therapeutic effects of transplanted hUC-MSCs.

**Conclusions**: We have identified miR-199a-5p as one of the mechanisms employed by hUC-MSCs to alleviate lupus disease associated pathologies in MRL/*lpr* mice, which is attributable for promoting splenic CD4+ T cell senescence through Sirt1/p53 pathway.

## Introduction

Systemic lupus erythematosus (SLE) is a chronic autoimmune and potentially fatal disease characterized by activation and proliferation of auto-reactive T cells and B cells with production of autoantibodies against nuclear and endogenous antigens 1. Current treatments for lupus are limited to anti-inflammatory and steroids synthetic cortisone medications with side effects, and often do not lead to improved outcomes for severe patients. Our previous studies demonstrated that human umbilical cord-derived mesenchymal stem cells (hUC-MSCs) alleviate the SLE disease both in humans and experimental animal models by immunomodulation, including regulating the Treg/Th17 balance, inhibiting T follicular helper cell expansion and so on 2-4. MSCs are widely known to suppress T cell responses through cell-cell contact and secretion of anti-inflammatory factors. As part of the mechanisms involved in this process, MSCs also inhibit T-cell proliferation by inducing T-cell unresponsiveness, arresting the cell cycle in the G1/G0 phase and promoting premature senescence 5, 6. However, the molecular pathways whereby MSCs induce T-cell-cycle arrest and suppress proliferation still await investigation.

Recently, cellular senescence, a form of cell cycle arrest, has emerged as an important mechanism for orchestrating immune cells during homeostasis and active immunity 7. It can be triggered by telomere shortening as well as a diversity of cellular stresses and signaling imbalances, also known as telomere-dependent senescence and telomere-independent senescence, respectively 8. The expansion of CD28null senescent T cells has been associated with disease severity in lupus patients 9, 10. Inconsistent with the definition of classical cellular senescence, previous researches focused on CD28null T cells, which has been described as a heterogeneous population and proliferate upon certain type of stimulation 11, 12. To date, the relationship between CD28null T cells and SLE disease activity appears controversial. 13. Furthermore, the function of CD28null senescent T cells has positive or adverse effect depend on senescence inducer and surrounding microenvironment 13-15. All these intrigue us to investigate the role of hUC-MSCs induced T cell premature senescence in lupus and the molecular mechanism of senescence induction.

Cellular senescence usually relies on the activation of the cyclin-dependent kinase inhibitors p21 and p16, which are components of the tumor-suppressor pathways governed by the p53 8, 16. While p53 acts as a threshold factor of cell homeostasis and senescence 17, its key upstream regulator NAD-dependent protein deacetylase sirtuin-1 (Sirt1) is essentially responsible for preventing cell fate towards senescence 18, 19. Consequently, silencing or inhibiting Sirt1 leads to p53 activation, and subsequently accelerate or drive senescence 20. Moreover, microRNAs (miRNAs) have been shown to regulate the levels of p53-target genes and regulators in senescence induction 21.

Employing the above-mentioned studies and considering the fact that MSCs communicate with surrounding immune cells by releasing miRNAs 22, we hypothesized that hUC-MSCs regulate target genes in signaling pathways of senescence via miRNAs may account for the therapeutic effect of hUC-MSCs in lupus. To test this hypothesis, we utilized the MRL/*lpr* mice to model SLE in human. MRL/*lpr* mice can reproduce key clinical pathologies of SLE and more importantly, the hyperactivation and pathogenicity of CD4+ T cell make this mouse model suitable for investigation of senescence-inducing therapy 23-25.

On the above basis, we postulated that improved understanding of the molecular mechanisms used in the generation of T cell telomere-independent senescence, including their functional alterations in SLE, could present novel approaches to normalizing T-cell function and consequently down regulating disease processes. In this study, we present evidence that hUC-MSCs increase senescence of splenic CD4+ T cells through regulating Sirt1/p53 via miR-199a-5p.

## Materials and methods

### Mice

Female MRL/*lpr* and ICR mice were purchased from Shanghai SLAC Laboratory Animal Co. Ltd. (Shanghai, China). Mice were housed under specific-pathogen-free conditions in the animal center of the Affiliated Drum Tower Hospital, Medical School of Nanjing University. All experimental animal protocols were approved by the Committee of Experimental Animal Administration of the Affiliated Drum Tower Hospital, Medical School of Nanjing University.

### Isolation, culture and identification of hUC-MSCs

The study protocol was approved by the Ethics Committee of the Affiliated Drum Tower Hospital, Medical School of Nanjing University. Umbilical cord specimen was collected from a 29-years-old parturient after taking signed informed consent forms. hUC-MSCs were prepared as previously described 26, 27, and cultured in DMEM/F12 supplemented with 10% fetal bovine serum and 100 U/ml penicillin/streptomycin (Gibco, Life Technologies, Grand Island, NY, USA) until confluent. The adherent cells were detached by 0.25% Trypsin-EDTA (Gibco) and reseeded into new flasks for further expansion. All the cell cultures were maintained at 37 °C in a 5% CO_2_ humidified atmosphere. Flow cytometry analysis showed hUC-MSCs used in this study were positive for the surface staining of CD73, CD90 and CD105, but lacked CD34, CD45, CD14, CD19 and HLA-DR expression.

### hUC-MSCs transplantation

Female MRL/*lpr* mice were randomly divided into two groups (hUC-MSCs and PBS treatment, n = 5 per group) and transfused with 5 × 10^5^ hUC-MSCs or PBS, respectively, via the tail vein at 17-weeks old. After 4 weeks, all treated mice were sacrificed for further analysis. Wild type (WT) ICR female mice were used as controls (n = 5) 28, 29.

### Isolation and culture of CD4+ T cells with hUC-MSCs

Mononuclear spleen suspensions of 17- to -20 weeks-old female MRL/*lpr* mice were prepared by gentle disruption with cell strainers (Corning, 352360) and lysed erythrocytes by RBC Lysis Buffer. The CD4+ T cells were purified using immunomagnetic positive selection (EasySep™ mouse CD4+ positive selection Kit, 18952, Stemcell technologies, Canada). They were then co-cultured with hUC-MSCs for 48 h at ratios of 1:1, 10:1, and 50:1 (T cells: MSCs), in the presence of soluble anti-mouse CD3 (2 μg/ml) and anti-mouse CD28 (2 μg/ml) antibodies. In some experiments, a transwell system (0.4 μm pore size, Millipore) was used to block cell-cell contact.

### CD4+ T cell culture and Sirt1 treatment

The CD4+ T cells were treated with either 50 μM EX527, a specific Sirt1 inhibitor, or 5 μM SRT1720, a Sirt1 activator (both from Selleck, Houston, TX, USA) for 12 h prior to coculture with hUC-MSCs for 24 h or 48 h in the presence of soluble anti-mouse CD3 (2 μg/ml) and anti-mouse CD28 (2 μg/ml) antibodies. Cells treated with DMSO alone were used as controls.

### Histopathology examination

Kidneys obtained at sacrifice were divided along their longitudinal axis. One half was fixed in 10% formaldehyde and embedded in paraffin. Three-micrometer-thick sections were stained with hematoxylin-eosin (HE) for histopathology. The other half of the kidney was embedded in Tissue-Tec OCT medium, frozen in liquid nitrogen, and stored at -70 °C until section processed. Five-micrometer-thick frozen sections were fixed with 4% paraformaldehyde and blocked with 2% BSA prior to staining with goat anti-mouse IgG (1:150 dilutions; Abcam, Cambridge, UK), followed by FITC-labeled anti-goat antibodies. Sections were photographed using a fluorescence microscope fitted with a digital camera (Cannon Power shot G10, Cannon, Inc.). Histological scores of renal lesions were calculated. Mainly, the severity of glomerulonephritis was graded on a 0-4 scale as follows: 0, normal; 1, mild increase in mesangial cellularity and matrix; 2, moderate increase in mesangial cellularity and matrix, with thickening of the glomerular basement membrane (GBM); 3, focal endocapillary hyper cellularity with obliteration of capillary lumina and a substantial increase in the thickness and irregularity of the GBM; and 4, diffuse endocapillary hyper cellularity, segmental necrosis, crescents and hyalinized end-stage glomeruli 30. The immunofluorescence (IF) intensity within the peripheral glomerular capillary walls and mesangial region were scored on a scale from 0 to 3 (0 = none; 1 = weak; 2 = moderate; 3 = strong) 27. At least 10 glomeruli/section were analyzed.

### Enzyme-linked Immunosorbent Assay (ELISA)

Serum levels of ANA, anti-dsDNA antibody, and total IgG were measured using a Mouse ANA ELISA Kit (Alpha Diagnostic, USA), a Mouse anti-dsDNA ELISA Kit (Shibayagi, Japan), and a Mouse IgG total ELISA Kit (Fcmacs, China) respectively, according to the manufacturers' instructions.

### Capillary WES analyses

After immunomagnetic positive selection, CD4+ T cells were lysed and protein concentration was measured by BCA (Thermo Fisher). Equal amounts of proteins were taken and diluted with 0.1× sample buffer. Four parts of diluted samples and 1 part 5× Fluorescent Master mix were mixed and transferred to stacking gel. After blocking, anti-Sirt1 (1:25, Cell Signaling Technology, 9475) and anti-Beta-Tubulin (1:25, proteintech, 10094-1-AP) were probed. HRP-conjugated secondary antibodies and chemiluminescent substrate were dispensed into designated wells in an assay plate. A biotinylated ladder provided molecular weight standards for each assay. After plate loading, the separation electrophoresis and immunodetection steps take place in the fully automated capillary system (WES, Protein Simple, San Jose, CA).

### Western blotting

Rabbit antibodies to Sirt1 (9475), acetyl-p53 (Lys379) (Cell Signaling Technology), p53 (10442-1-AP) (Proteintech), p16 (ab51243), p21 (ab109199) (Abcam), glyceraldehyde 3-phosphate dehydrogenase (GAPDH) (Cell Signaling Technology) and Goat anti-Rabbit IgG (H+L) (SA00001-2) (Proteintech) were used for western blotting. CD4+ T cells were washed twice with PBS and lysed on ice for 30 min with 1× RIPA buffer (Cell Signaling Technology) containing 1% 100× protease/phosphatase inhibitor Cocktail (Cell Signaling Technology). Lysates were centrifuged at 12,000 g at 4 °C for 20 min, and the supernatants were subjected to sodium dodecyl sulfate-polyacrylamide gel electrophoresis. Proteins were then transferred to polyvinylidene fluoride membranes (Millipore), blocked for 1 h in 5% nonfat milk (in 10 mM Tris pH 7, 150 mM NaCl, 0.1% Tween 20, TBST), and then immunoblotted with indicated primary antibodies and appropriate horseradish peroxidase-conjugated secondary antibodies. The bands were visualized in a luminol-based detection system with piodophenol enhancement.

### Quantitative real-time reverse transcription-polymerase chain reaction (RT-PCR) analysis

Total cellular RNA was extracted using Trizol reagent (Invitrogen) and 1 ug RNA was used in RT reactions. cDNA was synthesized by Primer Script RT reagent Kit (TaKaRa). Sequences of the forward and reverse primers were: p16 5'-CCCAACGCCCCGAACT-3' and 5'-GCAGAAGAGCTGCTACGTGAA-3'; p21 5'-GAGCAGTTGCGTGACCGTTG-3' and 5'-GGGGAATCTTCAGGCCGCTC-3'; Sirt1 5'-TCTATGCTCGCCTTGCGGTTG-3' and 5'-CCTGCAACCTGCTCCAAGGT-3'; GAPDH 5'-TGGCCTTCCGTGTTCCTAC-3' and 5'-GAGTTGCTGTTGAAGTCGCA-3'. MiR-199a-5p was performed by poly(A)-tailed RT-PCR as previously described 31. MiR-199a-5p primers and the 3'primer were: 5'-CCCAGUGUUCAGACUACCUGUUC-3'; 5'-ATTCTAGAGGCCGAGGCGGCCGACATGT-3'. Sequences of the U6 forward and reverse primers were: 5'-CGCTTCGGCAGCACATATACTA-3'; 5'-CGCTTCACGAATTTGCGTGTC3'. A HiPure miRNA Kit (Magen) was used to isolate miRNAs from cultured media samples; a total of 1000 μl of cultured media was used for each isolation. A cel-miR-39-3p standard RNA was added to samples as an external reference control. For real-time PCR experiments, reactions containing SYBR Premix EX Taq (Takara), ROX Reference Dye (50; Takara), cDNA, and gene primers were run on a Step One Plus real-time PCR system and analyzed using Step One Software, version 2.1 (Applied Biosystems). Relative gene quantification was calculated by the 2ΔCt method and then normalized to the level of GAPDH, U6 or miR-39-3p.

### Transfection of CD4+ T cells with miR-199a-5p mimic or inhibitor

The CD4+ T cells were seeded at density of 6 × 10^5^ cells/well in a 12-well plate. The cells were then transfected with either miR-199a-5p mimic or inhibitor obtained from GenePharma (Shanghai, China) using Entranster^TM^-R4000 Transfection reagent from Engreen (Beijing, China). The transfected cells were cultured for 48-72 h in the presence of soluble anti-mouse CD3 (2 μg/ml) and anti-mouse CD28 (2 μg/ml) antibodies. The transfected cells were then collected and used for total RNA and protein isolation.

### Culture of CD4+ T cells with RNase

MRL/*lpr* splenic CD4+ T cells and hUC-MSCs were cultured alone or together in the presence or absence of RNase (Qiagen, final concentration: 5 µg/mL) using a transwell system. The cells were processed as described above after 48 h addition of RNase.

### Dual luciferase reporter assay

To confirm miR-199a-5p targeted 3'-UTR of Sirt1, we constructed the fragment of sirt1 3'-UTR, which contains the predicted miR-199a-5p binding sites into pmirGLO Luciferase reporter vector (Promega, Madison, WI, USA). For the control, we introduced 507 A > T and 511 U > A point mutations located within the seed region of miR-199a-5p binding sites in Sirt1 3'UTR. Site-directed mutagenesis in the seed sequence of miR-199a binding site was introduced using In-Fusion cloning kit (Clontech, Mountain View, CA, USA) as per manufacturer's protocol. Dual-Luciferase Reporter Assay System was applied for luciferase reporter assay (Promega). HEK 293T cells were simultaneously transfected with WT or mutant (mut) type 3'-UTR Sirt1 and miR-199a-5p mimic or negative control (NC) mimic. Luciferase activity was detected using CytoFLEX Flow Cytomerter (Beckman Coulter). Firefly luciferase activity was normalized to renilla luciferase activity. The ratio of Sirt1 3'-UTR to NC mimic was set as 1; therefore, the results were presented as the fold changes relative to control.

### MiR-199a-5p agomir treatment in MRL/*lpr* mice

The miR-199a-5p agomir and agomir negative control (nc) were synthesized by RiboBio (Guangzhou, China) (n = 6 per group). Each mouse received a tail vein injection of either miR-199a-5p agomir or agomir nc, at a dose of 20 nmol in 0.2 ml saline every 4 days for 4 weeks and sacrificed 48 h after the last injection. Serum, spleens, lymph nodes and kidneys were harvested. The splenic CD4+ T cells were purified using immunomagnetic positive selection (EasySep™ mouse CD4+ positive selection Kit, 18952, Stemcell technologies, Canada).

### Flow cytometry

Single cell suspensions were prepared from the spleen by standard procedures. Fixable viability dye eFlour506 (ebioscience) were used to exclude dead cells. Flurochrome-conjugated antibodies from Biolegend were used in dilutions according to manufactures' instructions, which includes APC/Cyanine7 anti-mouse CD45, FITC anti-mouse CD3e, BV 711 anti-mouse CD4, PE anti-mouse CD27, BV421 anti-mouse CD28, APC/Cyanine7 anti-mouse CD45, FITC anti-mouse CD3e, BV510 anti-mouse CD8, BV711 anti-mouse CD44, BV605 anti-mouse CD62L, PE anti-mouse Foxp3, APC anti-mouse CD25. Foxp3 staining used Foxp3/Transcription Factor Staining Buffer (ebioscience) as recommended. Fortessa (BD biosciences) were used for acquisition and Flowjo (10.4) for analysis. Gating strategies for flow cytometry analysis are detailed in Supplementary information, Figure S1.

### Statistical analysis

The data were expressed as mean ± standard error of mean and analyzed with Prism 5 (GraphPad Software). Student's *t* test was used to analyze significance between two groups and comparisons among more than two groups were analyzed using one-way ANOVA. The value of *p* < 0.05 was considered statistically significant.

## Results

### hUC-MSCs ameliorate disease progression of MRL*/lpr* mice

**Figure 1:**
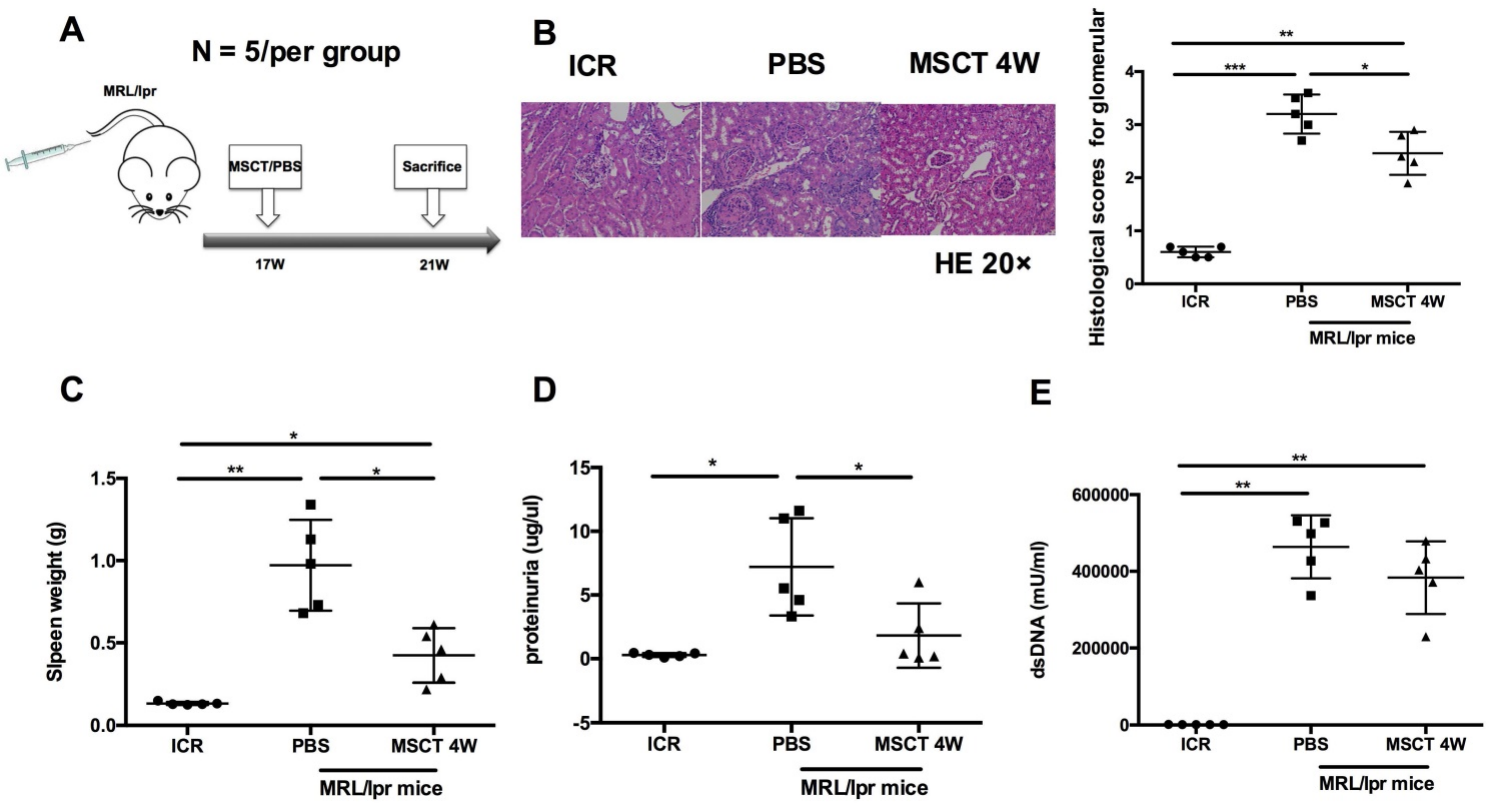
hUC-MSCs ameliorate disease of MRL/*lpr* mice. (A) Experimental outline describing the use of hUC-MSCs to treat MRL/*lpr* mice (n = 5 per group). Kidney lesion (B), spleen weight (C), proteinuria (D) and serum anti-dsDNA antibody (E) levels in WT mice, PBS-treated MRL/*lpr* mice and hUC-MSCs-treated MRL/*lpr* mice. *p < 0.05; **p < 0.01; ***p < 0.001.

To assess the therapeutic effect on the lupus syndrome of MRL/*lpr* mice, we treated MRL*/lpr* mice with 5 × 10^5^ hUC-MSCs or PBS starting at 17 weeks of age and sacrificed all mice at 21 weeks of age (Figure 1A). Compared to PBS-treated mice, the kidney lesions of hUC-MSC-treated mice were significantly reduced (Figure 1B). In addition, the spleen weight and proteinuria were much lower after hUC-MSCs treatment compared to the PBS-treated MRL/*lpr* mice (Figure 1C-D). Serum anti-dsDNA antibody levels were lower in the hUC-MSCs-treated group, but did not reach statistical significance (Figure 1E). The data indicate that hUC-MSCs ameliorate lupus disease in MRL*/lpr* mice.

### hUC-MSCs increase senescence of splenic CD4+ T cells through Sirt1/p53 signaling in MRL/*lpr* mice *in vivo*

**Figure 2:**
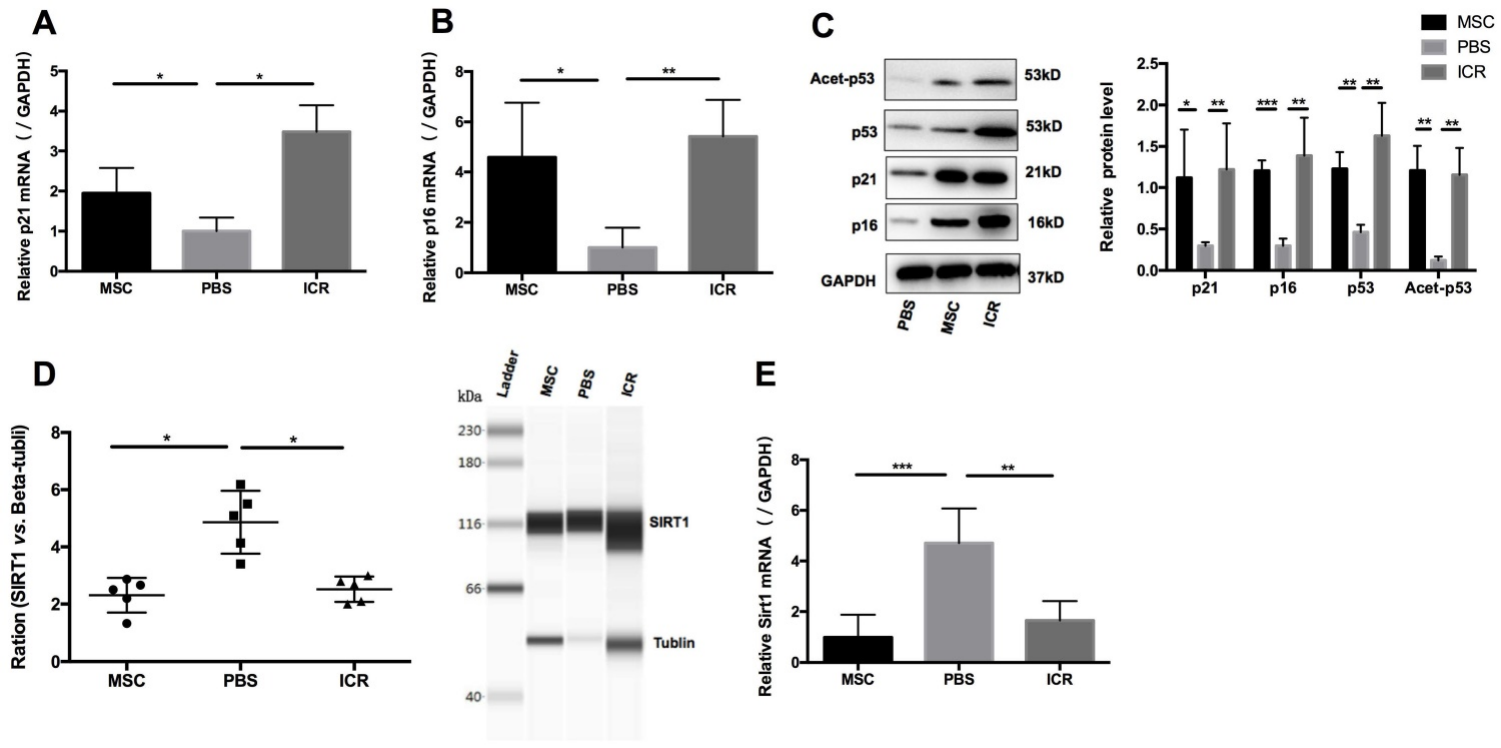
hUC-MSCs treatment normalizes markers of senescence in MRL/*lpr* mice splenic CD4+ T cells *in vivo.* (A, B) qPCR analysis showing the expression levels of p21, p16 in WT mice, PBS-treated MRL/*lpr* mice and hUC-MSCs-treated MRL/*lpr* mice. (C) Representative western blot showing the expression levels of p21, p16, p53 and acetylation of p53 in WT mice, PBS-treated MRL/*lpr* mice and hUC-MSCs-treated MRL/*lpr* mice. (D, E) Capillary WES and qPCR analysis showing the expression of Sirt1 in WT mice, PBS-treated MRL/*lpr* mice and hUC-MSCs-treated MRL/*lpr* mice. GAPDH was used as a protein loading control. *p < 0.05; **p < 0.01; ***p < 0.001.

We postulated that a potential mechanism of the therapeutic efficacy of hUC-MSCs in MRL/*lpr* mice could be through suppression of the accumulation of CD4+ T cells by enhancing their senescence. To test this, we isolated mononuclear cells from splenic cell suspension and then purified CD4+ T cells for RNA and protein preparation. We measured levels of p21 and p16 as markers of premature senescence. Our analysis showed that the mRNA and protein levels of p21 and p16 were significantly reduced in MRL/*lpr* mice compared to the WT control group, meanwhile, an increase in p21 and p16 mRNA and protein expression were seen in hUC-MSCs treatment group compared to PBS-treated MRL/*lpr* mice (Figure 2A-C). We also found that MRL/*lpr* splenic CD4+ T cells had increased Sirt1 levels and decreased acetylation of p53 at Lys-379 compared to the WT mice, while treatment with hUC-MSCs was able to reverse the changes by decreasing Sirt1 and increasing p53 and acetylation of p53 (Figure 2C-E). With the immunostaining CD45/CD3e/CD4/CD27/CD28, the senescent T cells can be identified as intermediate differentiation CD27-CD28+ population and highly differentiated CD27-CD28- population 32. There was a significant difference between two groups to the proportion of CD27-CD28+ cells, and an increasing trend toward CD27-CD28- cells in hUC-MSCs group (Supplementary information, Figure S2). Taken together, these results suggest that the senescence of splenic CD4+ T cells in MRL/*lpr* mice could be defective because of altered Sirt1/p53 signaling, which might result in CD4+ T-cell hyper proliferation. Thus, the clinical effects of hUC-MSCs could be associated with the restoration of senescence pathways in splenic CD4+ T cells.

### hUC-MSCs increase senescence of MRL/*lpr* splenic CD4+ T cells through Sirt1 *in vitro*

**Figure 3:**
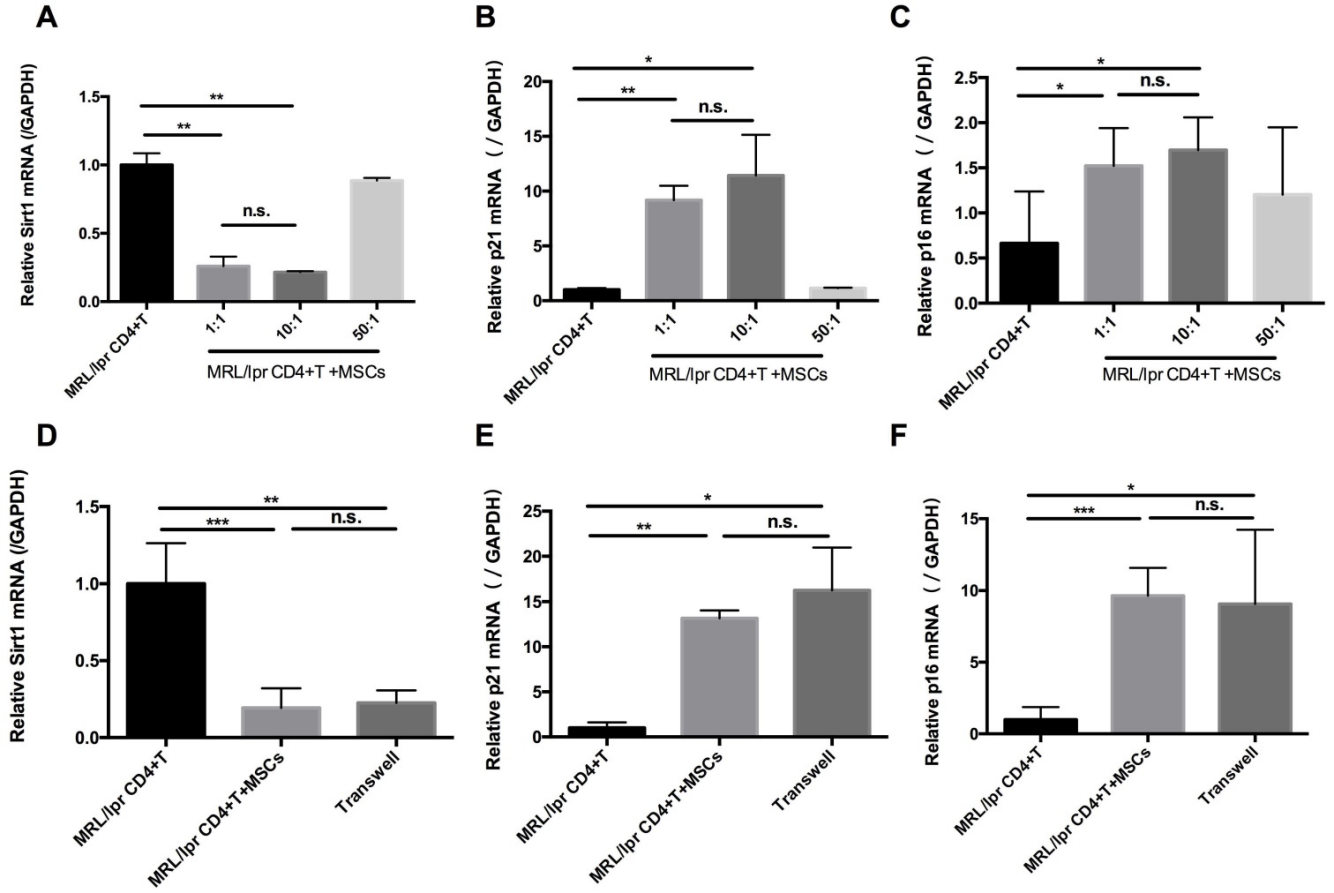
hUC-MSCs increase markers of senescence in splenic CD4+ T cells *in vitro*. (A-C) Splenic CD4+ T cells from MRL*/lpr* mice were cultured alone or with hUC-MSCs at ratios of 1:1, 10:1, or 50:1 for 48 h. (D-F) In further experiments, MRL/*lpr* splenic CD4+ T cells and hUC-MSCs (10:1) were cultured separated by transwells. Sirt1 (A, D), p21 (B, E) and p16 (C, F) RNA levels of CD4+ T cells were quantitated by qPCR. All experimental data were verified in at least two independent experiments. *p < 0.05; **p < 0.01; ***p < 0.001; n.s., not significant.

In parallel with the *in vivo* experiments, we determined the effects of hUC-MSCs on T cell senescence *in vitro*. Splenic CD4+ T cells from MRL/*lpr* mice were co-cultured with hUC-MSCs at different ratios (CD4+ T cells: MSCs: 1:1, 10:1, 50:1) for 48 h. We found that hUC-MSCs significantly up-regulated the levels of p21 and p16, but down-regulated Sirt1 levels in a dose-dependent manner (Figure 3A-C). Moreover, the modulation of cell senescence was not dependent on cell-cell contact, as similar results were found when the cultured CD4+ T cells and MSCs were separated by a transwell system (Figure 3D-F). These data suggest that soluble factors mediate the regulatory effects of hUC-MSCs on splenic CD4+ T cell senescence in MRL/*lpr* mice.

### Sirt1 is required for hUC-MSCs-mediated senescence of CD4+ T cells in MRL/*lpr* mice

**Figure 4:**
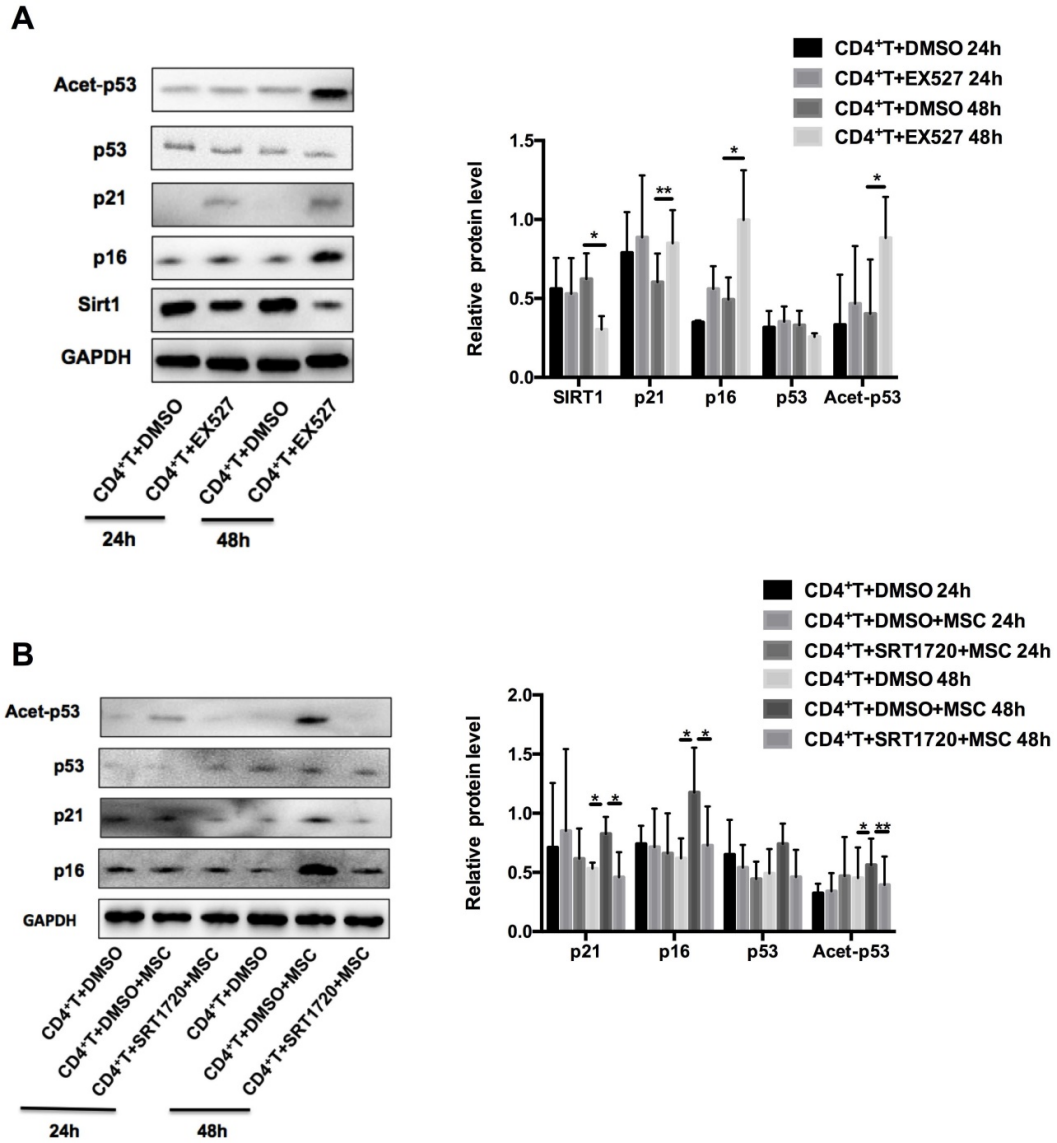
Sirt1 is a mediator of hUC-MSCs increasing senescence of splenic CD4+ T cells in MRL/*lpr* mice. (A) Western blotting showing the expression of Sirt1, p21, p16, p53 and acetylation of p53 in EX527 and DMSO treated MRL/*lpr* splenic CD4+ T cells. (B) MRL/*lpr* splenic CD4+ T cells were pretreated with SRT1720 12 h before exposed to hUC-MSCs for 24-48 h, western blotting showing the expression of p21, p16, p53 and acetylation of p53. GAPDH was used as a protein loading control. All experimental data were verified in at least three independent experiments. *p < 0.05; **p < 0.01.

Next, we examined whether Sirt1 is sufficient and necessary for hUC-MSCs to enhance the senescence of MRL/*lpr* splenic CD4+ T cells. We found that pretreatment of MRL/*lpr* CD4+ T cells with the Sirt1 inhibitor-EX527 attenuated expression of Sirt1 and increased expression p21 and p16, as well as the acetylation of p53 (Figure 4A). Conversely, pretreatment of T cells with a selective activator of Sirt1, SRT1720, inhibited the senescence induced by hUC-MSCs (Figure 4B). Collectively, these data reveal that Sirt1 is a key mediator whereby hUC-MSCs increase senescence of splenic CD4+ T cells in MRL/*lpr* mice.

### hUC-MSCs regulate Sirt1/p53 signaling in MRL/*lpr* splenic CD4+ T cells through transferring miR-199a-5p

**Figure 5:**
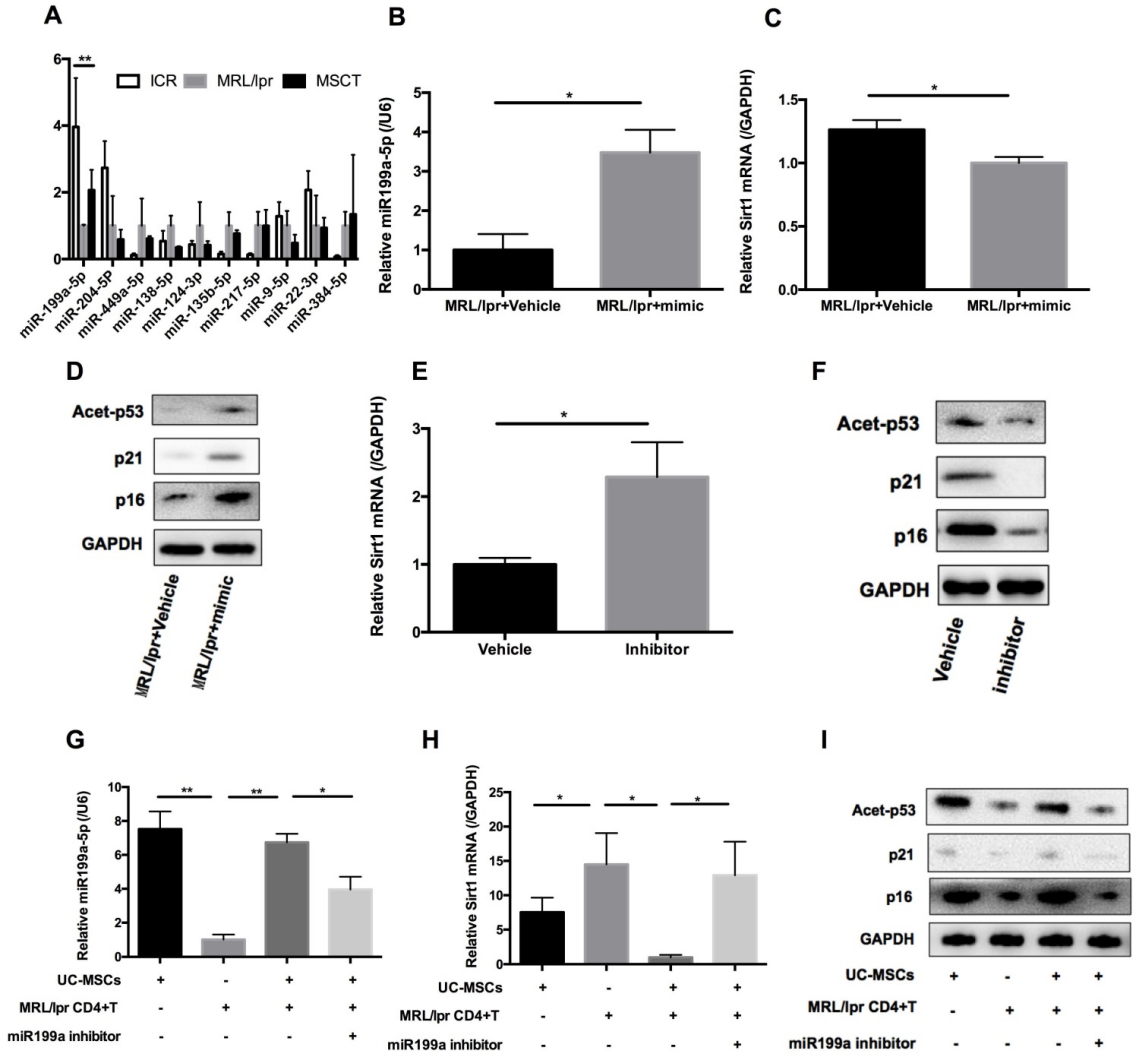
hUC-MSCs induced miR-199a-5p can increase MRL/*lpr* splenic CD4+ T cell senescence. (A) qPCR analysis of the levels of ten potential miRNAs in WT mice, PBS-treated MRL/*lpr* mice and hUC-MSCs-treated MRL/*lpr* mice splenic CD4+ T cells. (B-D) qPCR and western blotting analysis of the levels of miR-199a-5p, Sirt1, p21, p16 and acetyl-p53 in vehicle and miR-199a-5p mimic-treated MRL/*lpr* splenic CD4+ T cells. (E-F) qPCR and western blotting analysis of the levels of Sirt1, p21, p16 and acetyl-p53 in vehicle and miR-199a-5p inhibitor-treated WT splenic CD4+ T cells. (G-I) MRL/*lpr* splenic CD4+ T cells and hUC-MSCs were cultured alone or together in the presence or absence of miR-199a inhibitor using a transwell system. MiR-199a-5p, Sirt1, p21, p16 and acetyl-p53 were quantified in hUC-MSCs (the first bar) or splenic CD4+ T cells (the last three bars). GAPDH was used as a protein loading control. All experimental data were verified in at least two independent experiments. *p < 0.05; **p < 0.01.

hUC-MSCs are able to transfer miRNAs, which are small non-coding RNAs (~22 nucleotides) capable of silencing gene expression at a post-transcriptional level, to surrounding cells in cancer and autoimmune disease models 33. We therefore considered whether miRNAs could be involved in hUC-MSCs-mediated regulation of Sirt1 expression in splenic CD4+ T cells. We used software available online, Target scan, to identify 10 miRNAs that would be predicted computationally to target Sirt1 gene expression (Supplementary information, Table S1-2). qPCR analysis showed that of these 10 miRNAs, only miR-199a-5p was significantly decreased in MRL*/lpr* splenic CD4+ T cells (Figure 5A). Furthermore, miR-199a-5p was the only tested miRNA to show increased expression after hUC-MSCs treatment (Figure 5A). According to bioinformatic prediction, we also designed luciferase experiment to provide evidence of the direct interaction between the miR-199a-5p and Sirt1 mRNA (Supplementary information, Figure S3). In addition, we demonstrated a functional role of miR-199a-5p by showing that a miR-199a-5p mimic downregulated and a miR-199a-5p inhibitor upregulated Sirt1 expression in splenic CD4+ T cells *in vitro* respectively (Supplementary information, Figure S4). In fact, the miR-199a-5p mimic could not only increased the levels of miR-199a-5p and decreased Sirt1 expression in MRL/*lpr* splenic CD4+ T cells (Figure 5B-C), but also elevated expression of the senescence markers p21, p16 and acetyl-p53 (Figure 5D). Conversely, miR-199a-5p inhibitor treatment elevated the expression of Sirt1 in WT splenic CD4+ T cells (Figure 5E), and decreased the senescence markers p21, p16 and acetyl-p53 (Figure 5F). Finally, hUC-MSCs showed up to an increase in the production of miR-199a-5p as compared to MRL/*lpr* splenic CD4+ T cells, indicating that hUC-MSCs is the source of the key regulator miR-199a-5p (Figure 5G). Co-culture of hUC-MSCs with MRL/*lpr* splenic CD4+ T cells in a transwell system for 48 h showed increased intracellular miR-199a-5p along with decreased Sirt1 gene expression and restored expression levels of senescence markers in the CD4+ T cells, while miR-199a-5p inhibitor treatment remained depressed status (Figure 5G-I). The transwell cultures had also been undertaken with addition of RNase to the medium (Supplementary information, Figure S5). In addition, presence of miR-199a-5p in the supernatant from hUC-MSCs cultures was verified (Supplementary information, Figure S6). Collectively, the data suggest that hUC-MSCs increase splenic CD4+ T cell senescence through miR-199a-5p-mediated downregulation of Sirt1 and subsequent reduction of p53 deacetylation.

### MiR-199a-5p ameliorates disease in MRL/*lpr* mice

**Figure 6:**
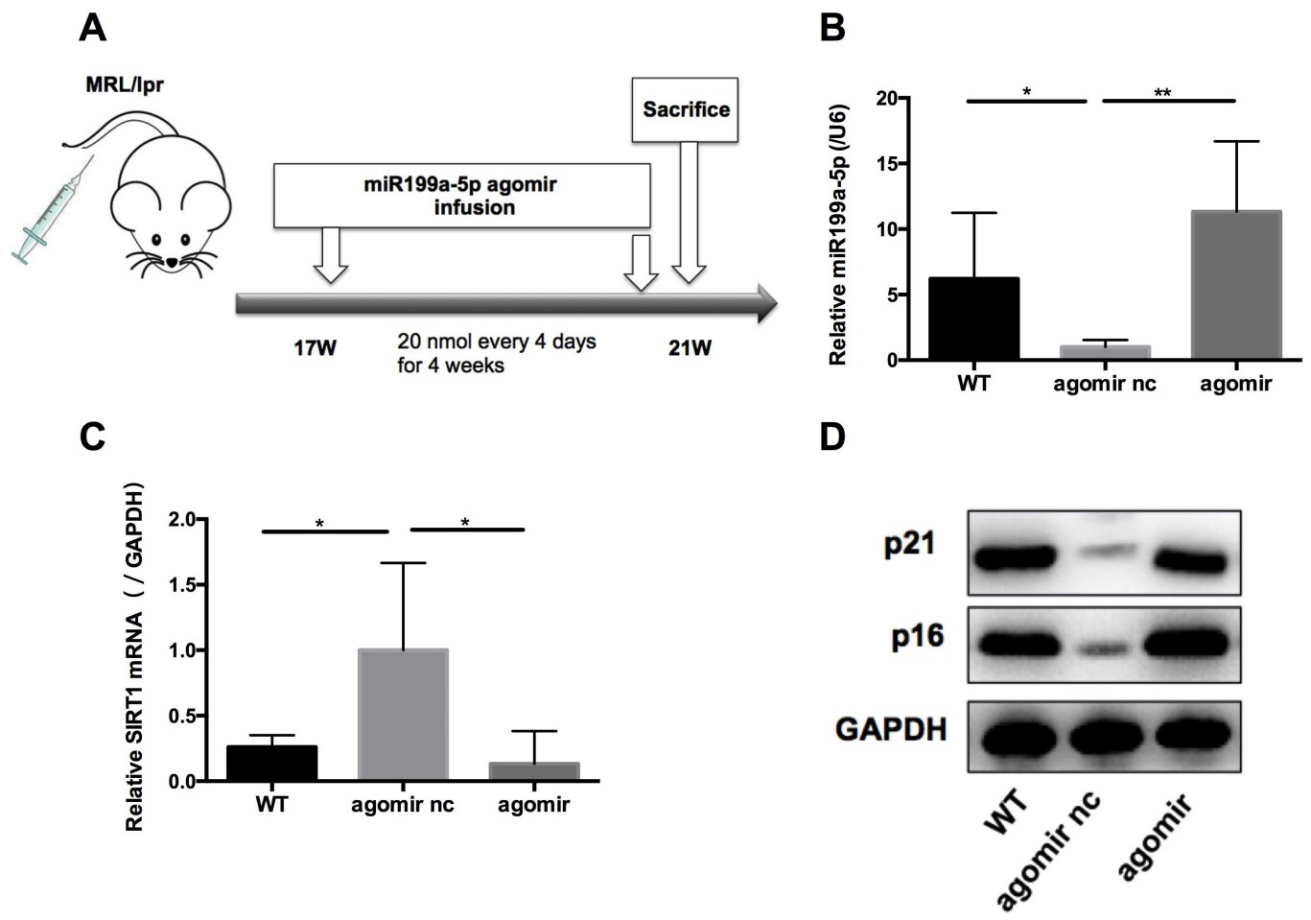
MiR-199a-5p agomir treatment increases senescence of MRL/*lpr* splenic CD4+ T cells. (A) Experimental outline. CD4+ T cells were harvested to measures miR-199a-5p (B), Sirt1 (C), p21 and p16 (D). GAPDH was used as a protein loading control. (n = 6 per group). *p < 0.05; **p < 0.01.

We next assessed the effects of miR-199a-5p on MRL*/lpr* mice* in vivo.* 17-weeks old MRL*/lpr* mice were injected with 20 nmol miR-199a-5p agomir or control (agomir nc) every 4 days for 4 weeks (Figure 6A). 48 h after the last injection, mice were sacrificed and splenic CD4+ T cells were isolated for examination. The miR-199a-5p agomir-treated group showed elevated levels of miR-199a-5p (Figure 6B), reduced expression of Sirt1 (Figure 6C), and marked up regulation of the senescence markers p21 and p16 compared to control treated cells (Figure 6D)*.* Moreover, miR-199a-5p agomir-treated group showed increased CD27-CD28+ cells and CD27-CD28- cells compared to control group (Supplementary information, Figure S7). The *in vivo* data suggest that hUC-MSCs increase MRL*/lpr* splenic CD4+ T cell senescence through miR-199a-5p.

**Figure 7:**
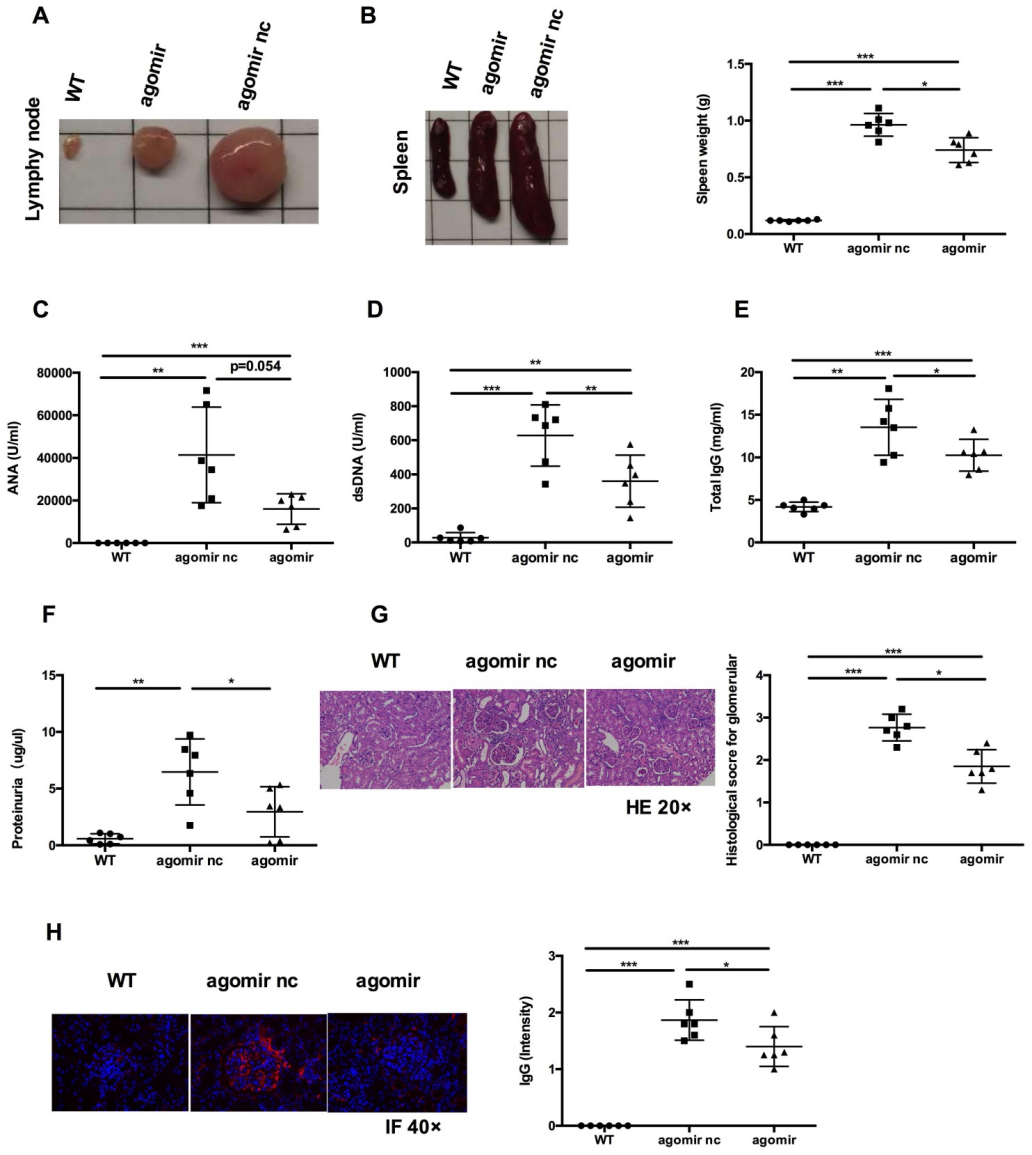
MiR-199a-5p agomir treatment ameliorates disease phenotype and immune disorder in MRL/*lpr* mice. MiR-199a-5p treatment reduced lymph node size (A), spleen index (B), serum levels of ANA (C), anti-dsDNA antibody (D), total IgG (E) and proteinuria (F) in MRL/*lpr* mice. (G, H) Representative images of renal pathology and immunohistochemical analysis after treatment with miR-199a-5p agomir. *p < 0.05; **p < 0.01; ***p < 0.001.

Next, we examined whether systemic administration of miR-199a-5p agomir rescued the lupus phenotype in MRL*/lpr* mice. When compared to agomir nc treatment group, miR-199a-5p agomir treatment significantly reduced the size of spleens and lymph nodes (Figures 7A-B) and decreased serum levels of anti-dsDNA antibody, IgG (Figure 7D-E). Serum ANA levels showed a tendency to decrease (Figure 7C). Renal impairments were also ameliorated in the miR-199a-5p agomir treated group, as shown by lower proteinuria (Figure 7F), reduced glomerular enlargement and hyper cellularity (Figure 7G) and less IgG deposition in the peripheral capillary loops (Figure 7H). Collectively, these data suggest that hUC-MSCs transplantation ameliorate disease phenotype in MRL*/lpr* mice through miR-199a-5p-mediated downregulation of Sirt1 signaling, and consequently increase senescence in splenic CD4+ T cells (see schematic diagram, Supplementary information, Figure S8).

## Discussion

Excessive T-cell proliferation with the subsequent clonal expansion is common immune imbalance phenomenon during the progression of SLE. As such, senescence of hyperactive T cells, accompanied with cell cycle arrest, could potentially reduce systemic inflammation and ameliorate SLE symptoms. In fact, researchers have already established the protective role of cellular senescence in tumor suppression 8, 34, wound healing 35, embryogenesis 36 and protection against tissue fibrosis 37. On the other hand, the negative effects of T cell senescence have been shown in tissue remodeling, aging and age-related disease 38. In brief, the effects of T cell senescence are context- and senescence inducer-dependent, and moreover, vary upon which subset that is being targeted. Given that MSCs transplantations inhibit T cells proliferation in SLE patients and mouse models 5, 39, we seek to establish connections between T cell premature senescence and beneficial effect of hUC-MSCs treatment. We prove the significant changes of senescence markers in splenic CD4+ T cells of MRL/*lpr* mice and then demonstrate that hUC-MSCs rescue lupus phenotype with induction of CD4+ T cell senescence. Our findings were in agreement with previous evidences of reduced p21 levels in lupus patient T cells and enhanced p21 expression for therapeutic potential in lupus-prone mice 40, 41. Taken together, these results suggest that the machinery of senescence induction in splenic CD4+ T cells of MRL/*lpr* mice is compromised and this might be associated with consequent hyper proliferation of CD4+ T cells.

Epigenetic modifications are characteristic of senescent changes. Taking into consideration the importance of histone modifying enzymes in fundamental processes such as cell proliferation and senescence, Sirt1 is one of the most important histone deacetylase catalyzing both histone and non-histone targets including p53 and FoxO proteins 19, 42. Sirt1 has been implicated in abnormal T-cell activation in the development of autoimmune syndrome, since Sirt1 deacetylates a variety of transcriptional factors and cofactors involved in apoptosis 43, 44. However, the impact of Sirt1 on T cell senescence is still unknown. In this study, we have demonstrated that MRL/*lpr* CD4+ T cells show increased Sirt1 expression and decreased acetylation of p53 *in vivo*. However, treatment with hUC-MSCs was able to decrease Sirt1 levels both *in vivo* and *in vitro*, and thus increase markers of senescence in splenic CD4+ T cells. Moreover, Sirt1 inhibitor could attenuate expression of Sirt1 and increase expression of acetylated p53, p21 and p16 in MRL/*lpr* splenic CD4+ T cells, while hUC-MSCs could not promote senescence when MRL/*lpr* splenic CD4+ T cells were pretreated with Sirt1 activator. These data suggest that Sirt1 is a mediator for hUC-MSCs induced senescence of splenic CD4+ T cells in MRL/*lpr* mice.

Our results show that soluble factors released from hUC-MSCs are responsible for promoting senescence by cell-to-cell contact-independent manner. Transferring miRNAs is one mean by which MSCs communicate with surrounding cells. A growing number of miRNAs were reported as key modulators of senescence in both human and mouse models 45, but their roles in regulating senescence in immune cells, especially in the context of SLE, are unclear. In the present study, we identified a novel target, miR-199a-5p, delivering from hUC-MSCs to splenic CD4+ T cells and assisting in T cell senescence. Through enhancing the expression of miR-199a-5p, the senescence of MRL/*lpr* splenic CD4+ T cells was increased. MiR-199a has been found to regulate osteogenic and chondrogenic differentiation of MSCs 46. In addition, regulatory role of miR-199a from adipose tissue-derived MSCs has also been reported to enhance the chemosensitivity in hepatocellular carcinoma through mTOR pathway 47. Notably, miRNAs have different target genes in various diseases and exert diverse regulatory functions 48, 49. In this study, we have shown that *in vivo* delivery of agomir produced mature miR-199a-5p in recipient cells and significantly reduced Sirt1 expression, resulting increased MRL/*lpr* splenic CD4+ T cell senescence. This result encouraged us to examine whether miR-199a-5p overexpression could rescue autoimmune disorders in MRL/*lpr* mice. Our data indeed indicated that miR-199a-5p agomir treatment successfully ameliorated disease phenotypes and restored immune homeostasis to some extent. However, it should be noted that its potential off-target effects needs to be explored in future study, in order to develop effective and safe miR-199a-5p-based gene therapy in clinical settings.

In the recent decade, therapies targeting cellular senescence pathways have been developed for a wide variety of diseases. Our study is based on the lupus-prone mouse model and support the therapeutic value of enhancing CD4+ T cell senescence in lupus mice via miR-199a-5p agomir. The clinical and epidemiological studies indicated that senescent CD4+ CD28null T cells were positively correlated with disease progression and renal damage in SLE patients 9, 10, but it remains to be clarified whether CD4+ CD28null T cells that expand as phenotypically and functionally heterogeneous population under chronic inflammation are truly senescent. In addition to CD28, terminally differentiated T cells in human can be better identified by expression of various other surface markers such as CD27, CD45R, CCR7 and CD57, resulting in a specific subset of “truly senescent” 32. Herein we identified CD27-CD28+ cells and CD27-CD28- cells in the MRL/*lpr* mouse that referenced as intermediate/highly differentiation population, which frequencies were higher in hUC-MSCs treated group and miR-199a-5p agomir-treated group when compared to controls. Furthermore, as compared with chronic pro-inflammatory state, hUC-MSCs infusion probably trigger and produce different premature senescent T cell subsets in a short-term. Therefore, further clinical studies are required to compare the hUC-MSCs induced senescent T cells and CD4+ CD28null T cells generated under SLE chronic inflammatory milieu.

In conclusion, we have shown here that hUC-MSCs increase splenic CD4+ T cell senescence through the Sirt1/p53 pathway via miR-199a-5p in lupus-prone MRL/*lpr* mice. These findings not only extend our knowledge of the immunoregulatory function of hUC-MSCs in SLE, but also offer miR-199a-5p as a potential biomarker or therapeutic target to further explore in the future.

## Supplementary Material

Supplementary figures and tables.

## Abbreviations

SLE: Systemic lupus erythematosus
hUC-MSCs: human umbilical cord-derived mesenchymal stem cells
Sirt1: sirtuin-1
miRNAs: microRNAs
WT: wild type
GBM: glomerular basement membrane
nc: negative control
HE: hematoxylin-eosin
IF: immunofluorescence
RT-PCR: Quantitative real-time reverse transcription-polymerase chain reaction
GAPDH: glyceraldehyde 3-phosphate dehydrogenase
