# Supplementary material for: Human umbilical cord-derived mesenchymal stem cell therapy ameliorates lupus through increasing CD4+ T cell senescence via MiR-199a-5p/Sirt1/p53 axis
Source: Theranostics. 2021 Jan 1;11(2):893–905. doi: 10.7150/thno.48080 (PMC7738872; doi:10.7150/thno.48080)
Supplement: Supplementary file 1 — Supplementary figures and tables. [file thnov11p0893s1.pdf]

Table S1. miRNAs hit on Sirt1

Rfam ID	Start	End	Alignment
mmu-miR-138-5p	35	42	5' ...AUUCAGGAAUUGCUC-CACCAGCA...<br>3' GCCGGACUAAGUGUUGUGGUCGA
mmu-miR-9-5p	345	352	5' ...GAACAGCUUAUCUAGACCAAAGA...<br>3' AGUAUGUCGAUCUAU--UGGUUUCU
mmu-miR-22-3p	475	482	5' ...UUUUCCAAAACUUGUGGCAGCUA...<br>3' UGUCAAGAAGUUGACCGUCGAA
mmu-miR-384-5p	72	78	5' ...AUGUCAAAAAAUGAAUGUUUACU...<br>3' UGUAACGGAUCCUUAACAAAUGU
mmu-miR-124-3p	1068	1075	5' ...UUUAAAAGCUUAGCUUGCCUUAA...<br>3' ACCGUAAGUGGCGCACGGAAUU
mmu-miR-135b-5p	304	310	5' ...UUUUAAAGUUUUCAAAAGCCAUU...<br>3' AGUGUAUCCUUACUUUUCGGUAU
mmu-miR-199a-5p	451	457	5' ...CAGUUAACUUUUUAAACACUGGC...<br>3' CUUGUCCAUCAGACUUGUGACCC
mmu-miR-217-5p	1355	1362	5' ...UGGCUACACUAAAGAAUGCAGUA...<br>3' AGGUCAGUCAAGGACUACGUCAU
mmu-miR-204-5p	325	332	5' ...UUGGAAUGUUAAUGUAAAGGGAA...<br>3' UCCGUAUCCUACUGUUUCCCUU
mmu-miR-449a-5p	781	787	5' ...AUCUUCACCACAAAU-ACUGCCAA...<br>3' UGGUCGAUUGUUAUGUGACGGU

Table S2. Gene primers used for Real-Time PCR

Genes	Primers
miR-138-5p	AGCUGGUGUUGUGAAUCAGGCCG
miR-9-5p	UCUUUGGUUAUCUAGCUGUAUGA
miR-22-3p	AAGCUGCCAGUUGAAGAACUGU
miR-384-5p	UGUAAACAAUUCCUAGGCAAUGU
miR-124-3p	UAAGGCACGCGGUGAAUGCC
miR-135b-5p	UAUGGCUUUUCAUUCCUAUGUGA
miR-217-5p	UACUGCAUCAGGAACUGACUGGA
miR-204-5p	UUCCCUUUGUCAUCCUAUGCCU
miR-449a-5p	UGGCAGUGUAUUGUUAGCUGGU

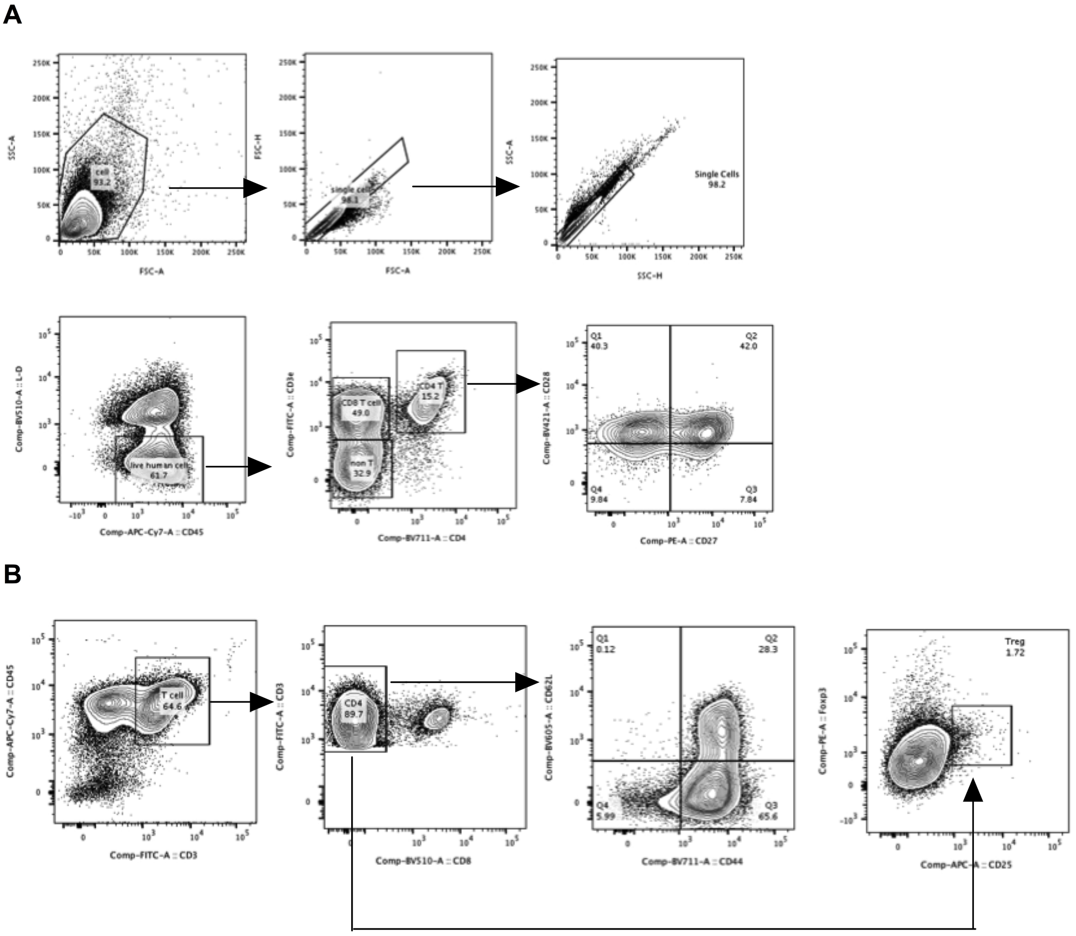

Figure S1. Gating strategy used to identify T-cells. (A). Gating strategy used to identify senescence T-cells. Gate 1: forward scatter area and side scatter area were used to enrich for intact cells. Gate 2 and 3: Side scatter pulse width and forward scatter pulse width were used to identify single cell events. A density plot with a tight gate provided the most stringent single cell gate. Gate 4: The BV510 channel was used to identify both dead cells and live cells using the leukocyte common antigen CD45. Gate 5: The CD4+ T cells were identified based on positive CD3 staining and positive CD4 staining. Gate 6: The differentiation population CD27-CD28+ and CD27-CD28- cells were identified based on CD27 staining and CD28 staining. (B). Gating strategy used to identify effector and regulatory T cells. Gate 1-3: as above described. Gate 4: The cells were identified using the leukocyte common antigen CD45 and T cell lineage restricted antigen CD3. Gate 5: The CD4+ T cells were subdivided into the main T cell subsets using CD3 and CD8. Gate 6: the presence of CD44 and CD62L were identified as shown. Gate 7: the indicated Foxp3+ CD25+ Treg cells are in the upper right gate.

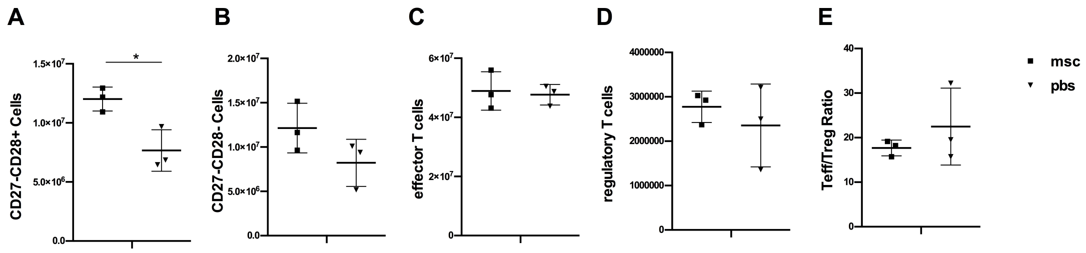

Figure S2. Comparisons of senescent T cells (A, B), effector T cells (C), regulatory T cells (D) and Teff/Treg ratio (E) between hUC-MSCs treated MRL/*lpr* group and PBS-treated group. Dot plots showing the absolute cell counts per mice.

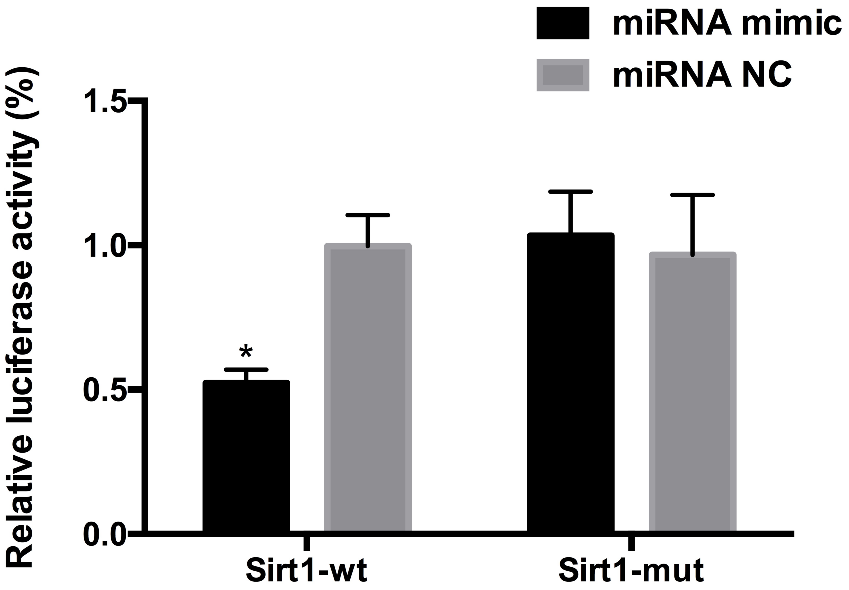

Figure S3. Effect of miR-199a-5p mimic and miR-199a-5p negative control (NC) on luciferase activity in HEK 293T cells transfected with either the wild type or mutant Sirt1 3'-UTR plasmid. *$p < 0.05$.

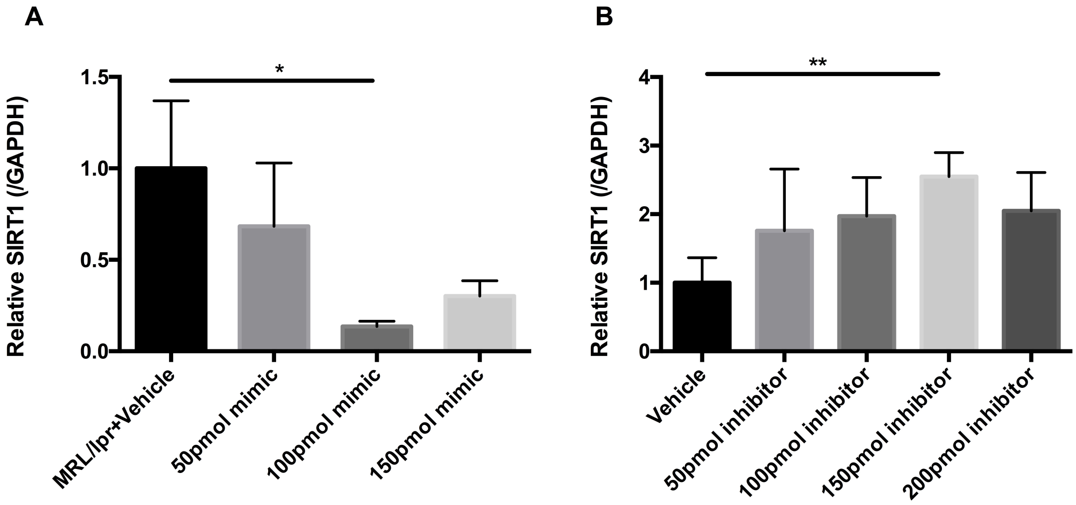

Figure S4. Dose responses of miR-199a-5p mimic and inhibitor. (A) Dose response assays showed that 100 pmole miR-99a-5p mimic was optimal for *in vitro* treatment in MRL/*lpr* splenic CD4+ T cells (B) Dose response assays showed that 150 pmole miR-199a-5p inhibitor was optimal for *in vitro* treatment in WT splenic CD4+ T cells. The results were representative of two independent experiments. *$p < 0.05$; **$p < 0.01$.

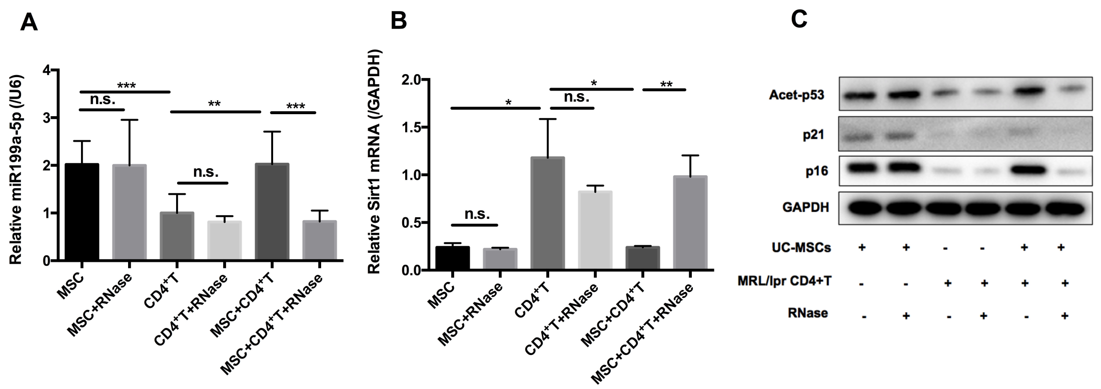

Figure S5. MRL/*lpr* splenic CD4+ T cells and hUC-MSCs were cultured alone or together in the presence or absence of RNase (Qiagen, final concentration: 5 μg/mL) using a transwell system for 48 h. qPCR and western blotting analysis of miR-199a-5p, Sirt1, p21, p16 and acetyl-p53 in vehicle and RNase-treated MRL/*lpr* splenic CD4+ T cells. GAPDH was used as a protein loading control. All experimental data were verified in three independent experiments. *$p < 0.05$; **$p < 0.01$; ***$p < 0.001$; n.s., not significant.

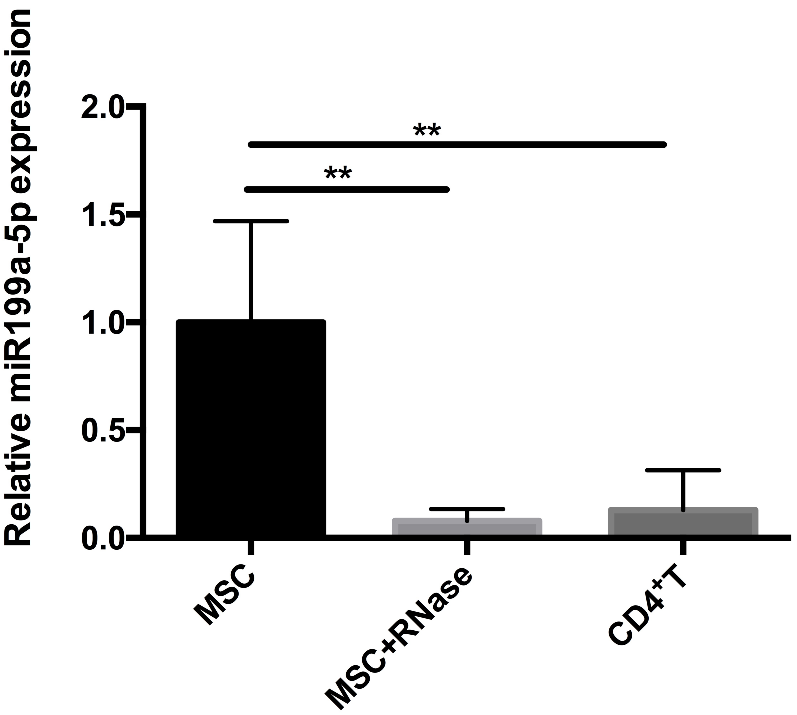

Figure S6. MiR-199a-5p expression levels in culture supernatant for hUC-MSCs. The results were representative of three independent experiments. **$p < 0.01$.

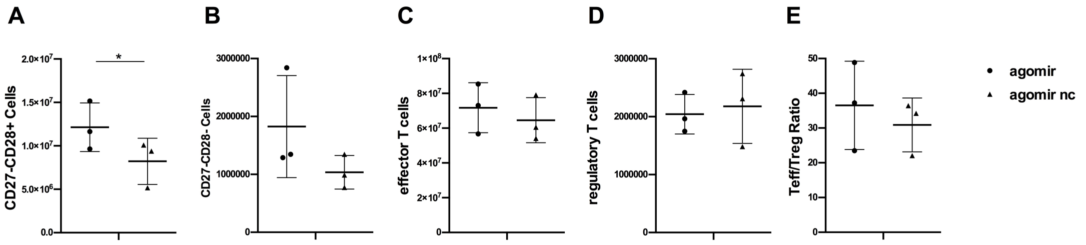

Figure S7. Comparisons of senescent T cells (A, B), effector T cells (C), regulatory T cells (D) and Teff/Treg ratio (E) between miR-199a-5p agomir treated MRL/*lpr* group and negative control. Dot plots showing the absolute cell counts per mice.

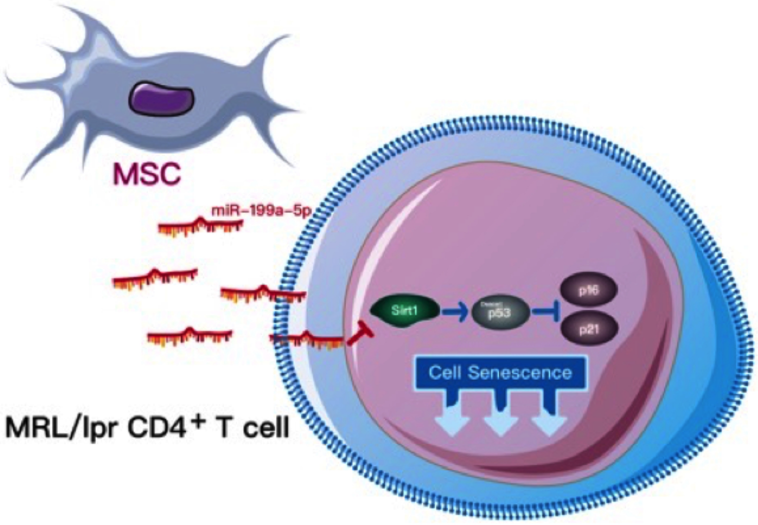

Figure S8. Potential mechanism for therapeutic effect of hUC-MSCs in murine lupus. hUC-MSC-transferring miR-199a-5p down regulates Sirt1 in CD4+ T cells, which in turn upregulate acetylation of p53 and expression of p21 and p16 to enhances cell senescence.
